# Potential and safety tests of egg drop syndrome candidate vaccine from Medan isolate, Indonesia

**DOI:** 10.14202/vetworld.2018.1637-1640

**Published:** 2018-11-29

**Authors:** Gusti Ayu Yuniati Kencana, Nyoman Suartha, I Made Kardena, Gusti Ayu Mayani Kristina Dewi, Arini Nurhandayani, Syamsidar, Kadek Karang Agustina

**Affiliations:** 1 Department of Virology, Faculty of Veterinary Medicine, Udayana University, Denpasar, Indonesia; 2 Department of Internal Medicine, Faculty of Veterinary Medicine, Udayana University, Denpasar, Indonesia; 3 Department of Pathology, Faculty of Veterinary Medicine, Udayana University, Denpasar, Indonesia; 4 Department of Poultry Science, Faculty Animal Husbandry, Udayana University, Denpasar, Indonesia; 5 PT. Sanbio Laboratories, Bogor, Jawa Barat, Indonesia; 6 Department of Public Health, Faculty of Veterinary Medicine, Udayana University, Denpasar, Indonesia

**Keywords:** hemagglutination, inhibition test, Medan isolate, safety and potential test, seed of egg drop syndrome vaccine

## Abstract

**Aim:**

The study was aimed to prepare and examine the potential and safety concerns of egg drop syndrome (EDS) vaccine candidate seed. The potential and safety trials of EDS Medan isolate vaccine need to be done before commercial scale of EDS vaccines are made.

**Materials and Methods:**

The safety test of EDS candidate vaccine was tested on 4-week-old specified pathogen-free chickens in an experimentally isolated enclosure.

**Results:**

The result of the safety test obtained 2^7.3^ hemagglutination inhibition (HI) unit of geometric mean titer antibody post-vaccination. However, the potency test of the EDS candidate vaccine was conducted on 17-week-old laying hens. Test results of the EDS potency vaccine in layer obtained antibody titer increased in every week of blood taking with average titer of antibody: Before vaccinated was 2^2.9^ HI unit, 1 week after vaccination was 2^3.7^ HI unit, 2 weeks post-vaccination was 2^5^ HI unit, and 3 weeks after vaccination was 2^7.3^ HI units. In contrast, decreasing trend was observed in control group (unvaccinated chicken).

**Conclusion:**

Serologically, the seed vaccine of EDS virus isolates from Medan was able to produce protective antibody titers starting in the 2^nd^ and 3^rd^ weeks post-vaccination.

## Introduction

Egg drop syndrome (EDS) is a disease caused by the duck adenovirus belonging to family Adenoviridae and genus of *Atadenovirus*. The virus was 1^st^ time discovered in 1976 in the Netherlands [1]. Therefore, it was called EDS - 1976 and later on abbreviated to EDS-76 [2]. Adenovirus particles are icosahedral in structure with 70-90 nm in diameter [3]. Virus particles are composed of 252 capsomers of triangular form. The genome of EDS 1976 is linear and composed of double-stranded deoxyribonucleic acid (DNA). Adenovirus is reported resistant to pH 3-9 but can be inactivated with 1:1000 formalin. Some strains of adenovirus can survive at 60-70°C for 30 min. The F1 strain can survive up to 56°C for 18 h. In Group I adenovirus, only F1 strain can agglutinate the red blood cells [4,5].

**Figure-1:**
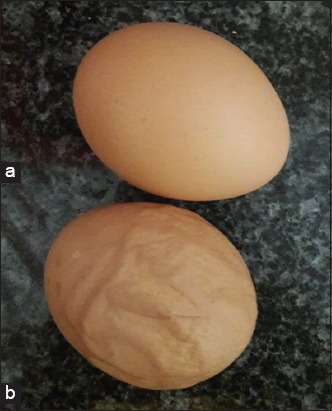
(a) Normal egg and (b) Egg drop syndrome egg abnormalities (personal archive).

EDS is a viral disease of layer birds, found mainly during peak production causing high economic losses [6,7]. EDS mainly attacks layers that are in the reproductive ages of 25-26 weeks [8]. Ducks and geese are reservoirs of the EDS virus and are the sources of virus transmissions through contaminated water [9]. The EDS disease is characterized by specific symptoms of decrease in the quality and quantity of eggs during peak production. Low egg quality is characterized by small size egg with mushy eggshell which can be easily broken (Figure-1) [10,11], which leads farmers to huge economic losses. EDS virus initially infected breeding farms and later on spread to other farms through infected eggs [12]. Adenovirus has become endemic in various parts of the world including Indonesia. It is a strategic infectious disease that must be eradicated [13]. EDS can be prevented by vaccination of layer before laying. In Indonesia, EDS vaccination has been adopted by several farmers, but EDS outbreak is still reported from layer birds [11].

For this purpose, isolation and characterization of EDS virus from field isolate are required. Isolate Medan used as seed vaccine taken from commercial poultry farms in Medan that shows the clinical symptoms of decreased egg production with a flabby shell [11]. With such an assumption, the vaccine made from local seed virus would be more appropriated if it used to prevent EDS epidemic in Indonesia. This study was carried out to test the potential and safety of vaccines derived from local isolates.

## Materials and Methods

### Ethical approval

This research was accepted in the Ethical Commission for the Use of Animals in Research and Education of the Faculty of Veterinary Medicine, Udayana University, Indonesia with Ref. No. 297a/KE-PH/VII/2017.

### EDS vaccine candidate

EDS isolates came from the city of Medan (Indonesia), which was taken from the field case of laying hens with EDS clinical symptoms. Samples of isolates were taken from the uterus and oviducts, isolated in 11-day-old spotted duck eggs. Inoculated for 3 days and then harvested, the allantoic liquid was tested by hemagglutination (HA) and polymerase chain reaction tests. The HA test results of the viral titers were 2^12^ HA units, and DNA amplification was 500 bp [11]. The EDS isolates of Medan were subsequently inactivated, emulsified, and used as EDS vaccine seeds. Safety test for EDS vaccine candidate was performed on specified pathogen-free (SPF) chickens in the experimental cage at PT. Sanbio Laboratories, while the potential test was done on commercial layer at the Faculty of Animal Husbandry, Udayana University.

The safety test of the EDS vaccine candidate was performed in 10 SPF chickens of 4-week-old vaccinated by injecting one dose in thigh muscles with EDS vaccine candidate. 10 other SPF chickens were used as controls. The content of EDS virus for vaccine candidates was made by conducting double-fold serial dilutions starting from titers 2^1^ to 2^20^ to be formulated into 2^15^ HA units as requirements for candidate vaccines based on Indonesian Medicine Federation standards. The 2^15^ HA units were calculated by the HA test. The vaccine manufacturing process begins with adding 70% of the oil phase (adjuvant) to inactivated EDS virus isolates into the mixing tank, followed by the addition of 30% water phase, emulsified to homogeneous. The volume of vaccine candidates injected into SPF chickens was 0.5 ml containing 2^15^ HA units, while the control was injected with 0.5 ml phosphate-buffered saline. Observation of the safety test of the EDS vaccine candidate was conducted for 2 weeks. Seed vaccine was said to be eligible if all the treated and control groups of the chickens remained alive and well during the study period.

The potency test of EDS vaccine candidate was done at the Faculty of Animal Husbandry, Udayana University. A total of 50 layers were used for the research purpose. Vaccination was performed on 18-week-old layers with one dose (0.5 ml) of intramuscular vaccine in thigh muscles. Blood sampling for serum was performed for 4 times: The 1^st^ time before vaccination and later on 3 times was performed every week after post-vaccination up to the 4^th^ week. Every day, the clinical symptoms are observed, and at the end of the study, all chickens were killed to observe their gross pathological changes.

The antibody titers were tested by HA reaction and inhibition-based serological test (HA/hemagglutination inhibition [HI]) [14-16]. These tests were performed at Virology Laboratory, Faculty of Veterinary Medicine, Udayana University. The antibody titers obtained were statistically analyzed using ANOVA test [17].

## Results and Discussion

The result of the research of Medan isolate showed that the seeds of EDS vaccine used in Medan isolate did not cause clinical symptoms in the chickens. Thus, it can be concluded that the EDS vaccine seed isolate Medan is safe to be used. Serologic test results with HA resistance from vaccinated SPF serum showed a mean of the titer of geometric mean titer antibody of 2^7.3^ with the lowest titer of 2^5^ HI unit, while the highest titer was 2^10^ HI unit. Unlike the antibody titer of non-vaccinated control chicken, the HA test results showed zero titer (Table-1). This proves that the Medan EDS virus isolates are safe to be used as EDS vaccine seeds.

**Table-1:** Antibody titer of SPF chickens post-vaccination of EDS seed vaccine.

Group	Total sample	Titer HI microtiter (Log_2_)												Mean (Log_2_)	Min (Log_2_)	Max (Log_2_)	GMT	Coefficient of the variation (%)

		EDS																
		0	1	2	3	4	5	6	7	8	9	10	11					
		1	2	4	8	16	32	64	128	256	512	1024	2048					
EDS vaccine	10	0					1	2	3	2	1	1		7.3	5	10	158	20
Control	10	0												0	0	0	1	0

SPF=Specific pathogen free, EDS=Egg drop syndrome, GMT=Geometric mean titter, HI=Hemagglutination inhibition

**Figure-2:**
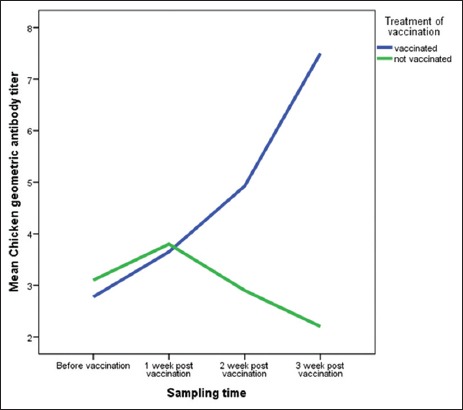
The increased titer of egg drop syndrome antibody in laying hens before and after vaccination.

Potency test of EDS vaccine seeds in commercial laying chickens vaccinated at 17 weeks, produced an antibody titer that increased in every week post-vaccination. Serologically, the EDS vaccine candidate of Medan isolates is eligible for EDS vaccine because it was able to produce antibody titer of 2^7.3^ HI units in layer vaccinated at 17 weeks (Figure-2). The antibody titer result was protective antibody titer because it was > 2^4^ HI units. Bidin *et al*. [18] reported on EDS-vaccinated chickens at 18 weeks of age to produce antibody titers of 16-256 HI units or 2^4^-2^8^ HI units in 96.7% of the samples. The increase of EDS antibody titer in this study is also similar to the results of vaccination with the commercial vaccine Shanvac ND-IB-EDS in commercial laying hen farms that were capable to trigger antibody titer of 2^7^ HI units for 3-week post-vaccination period [19].

The result of analysis variance showed that vaccine administration had a significant effect on chicken antibody titer (p<0.05). The time of blood taking had a significant effect on chicken antibody titer (p<0.05). There was also a significant association between timing of vaccination and serum collection (Table-2). While the antibody titer in the control group of chicken decreased during maintenance period. This may be due to the presence of maternal antibodies due to vaccination in the mother [20,21] and can also result from non-specific infections that reduce general immunity [22].

**Table-2:** Effects of the vaccination on antibody against EDS in the commercial laying chickens.

Source	Type III sum of squares	df	Mean square	F	Significant
Intercept					
Hypothesis	1994.425	1	1994.425	434.604	0.000
Error	220.275	48	4.589^a^		
Treatment					
Hypothesis	93.845	1	93.845	20.450	0.000
Error	220.275	48	4.589^a^		
Treatment*repetition					
Hypothesis	220.275	48	4.589	1.457	0.047
Error	453.575	144	3.150^b^		
Time					
Hypothesis	59.265	3	19.755	6.272	0.000
Error	453.575	144	3.150^b^		
Treatment*time					
Hypothesis	164.705	3	54.902	17.430	0.000
Error	453.575	144	3.150^b^		

Dependent variable: Antibody titer of laying chickens post-vaccination of EDS seed vaccine. EDS=Egg drop syndrome

Clinical observation results in post-vaccinated chickens with EDS isolate Medan did not experience any clinical sign. The chickens that got vaccination by EDS isolates Medan vaccine candidate produced eggs with normal in shape, size, and production. Unlike the infected chickens, EDS caused low-quality eggs such as thin eggshells and around 10-30% decline in egg production [23]. In experimental infections, respiratory distress occurs in the 3-4 days post-infection period with a 10-40% decrease in production, but no death was observed [9].

## Conclusion

Based on the results of the experimental test of Medan isolate EDS candidate vaccine in SPF chickens or in layers, it can be concluded that EDS vaccine is safe to be used as a vaccine against EDS. Seeds of EDS vaccine Medan isolates may trigger protective antibody formation from 2-week post-vaccination with antibody titers of 2^5^ HI units and 2^7.3^ HI units for 3-weeks post-vaccination. Further, research on EDS Medan vaccine isolates is needed to be used as EDS vaccine and its effect on production in commercial chicken farms. The challenge infection by virulent EDS virus post-vaccination is not done in the present study, but we wish to do it in the future.

## Authors’ Contributions

GAYK: Plan, conduct research, and write a script. NS: Perform serological tests and test vaccine potential. IMK: Potential test, data analysis, and write a script, GAMKD: Maintain and supervise experimental chickens. AN: Virus isolation and vaccine safety test. S: Virus isolation and vaccine safety test. KKA: Data analysis and write a script. All authors read and approved the final manuscript.
